# PD125 Safety, Efficacy, And Effectiveness Of Robotic Surgery In General And Digestive Surgery

**DOI:** 10.1017/S0266462324003623

**Published:** 2025-01-07

**Authors:** Jessica Ruiz-Baena, Joan Segur-Ferrer, Laia Ramos Masdeu, Maria-Dolors Estrada Sabadell, Rosa Maria Vivanco-Hidalgo

## Abstract

**Introduction:**

Robotic surgery (RS) is a minimally invasive surgical modality performed with the support of a console and mechanical arms that enable remote control. The advantages of RS are clear from the point of view of surgeons but remain unclear in terms of clinical results. We evaluated the safety, efficacy, and clinical effectiveness of RS compared with open or laparoscopic surgery.

**Methods:**

A systematic review of randomized controlled trials and systematic reviews with meta-analyses was conducted to assess RS in the following surgical procedures: Nissen fundoplication, Heller myotomy, cholecystectomy, rectopexy, splenectomy, pediatric Kasai portoenterostomy, and gastric banding. Outcomes of interest were related to safety (complications, blood loss, and risk of infection) and efficacy or effectiveness (length of hospital stay, quality of life [QoL], recovery, patient satisfaction, conversion to another technique, urinary function, and rates of mortality, readmission, reoperation, and esophageal perforation). The evidence quality was assessed with version two of the Cochrane risk-of-bias tool for randomized trials, AMSTAR 2, and GRADE.

**Results:**

Nissen fundoplication RS was similar to laparoscopy in terms of complication and conversion rates, recovery, and QoL. Heller myotomy RS reduced the rate of esophageal perforations but had similar perioperative blood loss and rates of mortality, conversion, and re-admission to laparoscopy. Cholecystectomy RS was similar to laparoscopy with respect to rates of readmission and complications, blood loss, and risk of infection. Rectopexy RS was similar to laparoscopy in terms of conversion, reoperation, and complications rates, blood loss, recovery, patient satisfaction, and QoL. Splenectomy RS decreased blood loss but was similar in risk of infection and rates of complications and conversion to laparoscopy. There were no studies that used open surgery as a comparator or evaluated RS in pediatric Kasai portoenterostomy or gastric banding.

**Conclusions:**

Current evidence on the safety, efficacy, and clinical effectiveness of RS in general or digestive surgical procedures is limited. Multicenter studies with follow-up beyond the immediate postoperative period are needed to evaluate patient outcomes.

